# Leveraging a Population-Based Dyadic Data Set to Promote Health Equity Among Chinese Americans

**DOI:** 10.1093/geroni/igab046.764

**Published:** 2021-12-17

**Authors:** XinQi Dong, Dexia Kong

## Abstract

Recognizing the central role of family-oriented values in Chinese culture, developing a family-based understanding of health and wellbeing in Chinese Americans is imperative. By linking two unique population-based datasets (one on Chinese older adults, and another on their corresponding adult children caregivers), the purpose of this symposium is to present interactive analyses of dyad-level data to achieve an interpersonal understanding of health outcomes of Chinese older adults and their adult children within the family context. Data were obtained from 807 Chinese older adults-adult children dyads by merging data from two epidemiological studies, namely the Population Study of ChINese Elderly in Chicago (the PINE study) and the PIETY study of corresponding adult children caregivers of PINE participants. Specifically, this symposium presents findings from five interconnected research projects. Session 1 provides an overview of study design and sample characteristics of the dyadic dataset. Session 2 examines the relationship between adult children’s endorsement of the filial piety value and older parents’ mental health outcomes. Session 3 investigates the level of congruence between older parents’ self-perceived mental health and adult children’s evaluation of their parents’ mental health. Session 4 investigates the extent to which depressive symptoms among older parents were associated with those of their adult children. Session 5 explores the relationship between older parents’ physical function and adult children’s perceived caregiving burden. Taken together, this symposium presents potential contributions of dyad-level analyses in advancing minority population health. Study findings have the potential to inform the development of family-centered intervention strategies targeting Chinese Americans.

